# 6,6′-Di-*tert*-butyl-2,2′-[1,2-phenyl­ene­bis(nitrilo­methyl­idyne)]diphenol

**DOI:** 10.1107/S1600536809048922

**Published:** 2009-11-21

**Authors:** Naser Eltaher Eltayeb, Siang Guan Teoh, Chin Sing Yeap, Hoong-Kun Fun, Rohana Adnan

**Affiliations:** a School of Chemical Science, Universiti Sains Malaysia, 11800 USM, Penang, Malaysia; b X-ray Crystallography Unit, School of Physics, Universiti Sains Malaysia, 11800 USM, Penang, Malaysia

## Abstract

The mol­ecule of the title Schiff base compound, C_28_H_32_N_2_O_2_, has a twisted geometry, the dihedral angles between the central benzene ring and the other two benzene rings being 29.12 (14) and 26.01 (14)°. Four intra­molecular C—H⋯O hydrogen bonds and two intra­molecular O—H⋯N hydrogen bonds stabilize the mol­ecular structure. In the crystal packing, mol­ecules are stacked along the *a* axis and stabilized by π–π inter­actions [centroid–centroid distance = 3.6724 (17) Å]. The crystal studied was found to be a non-merohedral twin, the refined ratio of twin components being 0.374 (5):0.626 (5).

## Related literature

For biological applications of Schiff base derivatives, see: Dao *et al.* (2000 ▶); Eltayeb & Ahmed (2005*a* ▶,*b* ▶); Karthikeyan *et al.* (2006 ▶); Sriram *et al.* (2006 ▶). For related structures, see: Eltayeb *et al.* (2007*a* ▶,*b* ▶). For the stability of the temperature controller used for the data collection, see: Cosier & Glazer (1986 ▶).
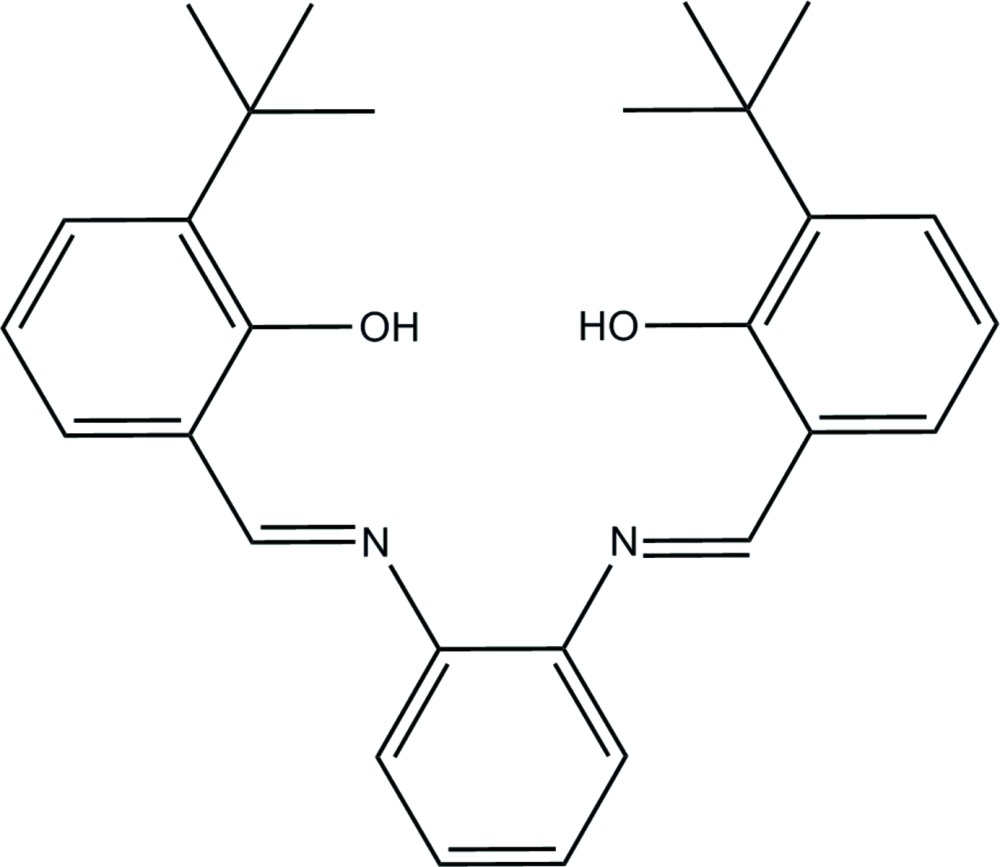


## Experimental

### 

#### Crystal data

C_28_H_32_N_2_O_2_
*M* *_r_* = 428.56
Triclinic, 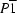

*a* = 6.8312 (9) Å
*b* = 13.9632 (16) Å
*c* = 14.0689 (15) Å
α = 116.615 (5)°
β = 99.068 (4)°
γ = 98.209 (4)°
*V* = 1149.6 (2) Å^3^
*Z* = 2
Mo *K*α radiation
μ = 0.08 mm^−1^
*T* = 100 K
0.87 × 0.20 × 0.05 mm

#### Data collection

Bruker SMART APEXII CCD area-detector diffractometer
Absorption correction: multi-scan (**SADABS**; Bruker, 2005 ▶) *T*_min_ = 0.936, *T*_max_ = 0.996
4021 measured reflections
4021 independent reflections
3241 reflections with *I* > 2σ(*I*)

#### Refinement

*R*[*F* ^2^ > 2σ(*F* ^2^)] = 0.059
*wR*(*F* ^2^) = 0.206
*S* = 1.19
4021 reflections
304 parameters
H atoms treated by a mixture of independent and constrained refinement
Δρ_max_ = 0.32 e Å^−3^
Δρ_min_ = −0.39 e Å^−3^

### 

Data collection: *APEX2* (Bruker, 2005 ▶); cell refinement: *SAINT* (Bruker, 2005 ▶); data reduction: *SAINT*; program(s) used to solve structure: *SHELXTL* (Sheldrick, 2008 ▶); program(s) used to refine structure: *SHELXTL*; molecular graphics: *SHELXTL*; software used to prepare material for publication: *SHELXTL* and *PLATON* (Spek, 2009 ▶).

## Supplementary Material

Crystal structure: contains datablocks global, I. DOI: 10.1107/S1600536809048922/lh2954sup1.cif

Structure factors: contains datablocks I. DOI: 10.1107/S1600536809048922/lh2954Isup2.hkl

Additional supplementary materials:  crystallographic information; 3D view; checkCIF report

## Figures and Tables

**Table 1:** Hydrogen-bond geometry (Å, °)

*D*—H⋯*A*	*D*—H	H⋯*A*	*D*⋯*A*	*D*—H⋯*A*
O2—H1*O*2⋯N2	0.91 (5)	1.73 (4)	2.584 (3)	156 (4)
O1—H1*O*1⋯N1	0.91 (5)	1.77 (6)	2.609 (3)	151 (5)
C22—H22*A*⋯O1	0.96	2.34	2.993 (3)	125
C23—H23*A*⋯O1	0.96	2.34	2.987 (4)	124
C26—H26*B*⋯O2	0.96	2.31	2.963 (4)	125
C27—H27*C*⋯O2	0.96	2.37	3.011 (4)	124

**Fig. 1.**
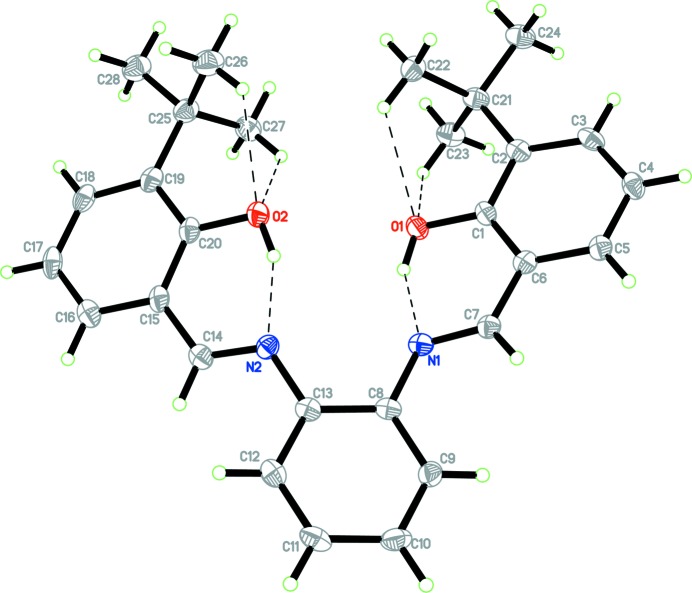
The molecular structure of the title compound with 50% probability ellipsoids for non-H atoms. Intramolecular hydrogen bonds are shown as dashed lines.

**Fig. 2.**
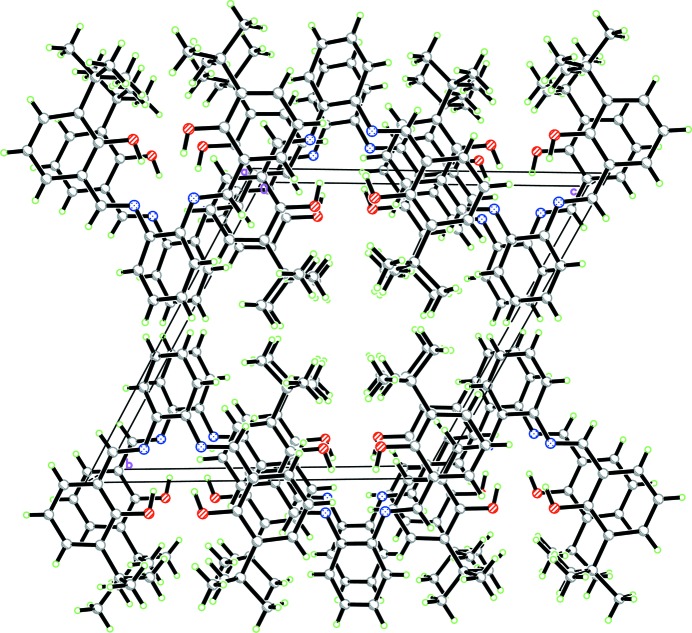
The crystal packing of the title compound, viewed along the a axis, showing how the molecules are stacked along the a axis.

## supplementary crystallographic information

### Comment

Schiff bases have received much attention because of their potential applications with some of these compounds exhibiting various pharmacological activities, as noted by their anticancer (Dao *et al.*, 2000), anti-HIV (Sriram *et al.*, 2006), antibacterial and antifungal (Karthikeyan *et al.*, 2006) properties. In addition, some of them may be used as analytical reagents for the determination of trace elements (Eltayeb & Ahmed, 2005*a*,b). Previously, we have reported the crystal structures of 2,2'-[1,2-phenylenebis(nitrilomethylidyne)]bis(5-methylphenol) (Eltayeb *et al.*, 2007*a*) and 6,6'-dimethyl-2,2'-[1,2-phenylenebis(nitrilomethylidyne)]diphenol (Eltayeb *et al.*, 2007*b*). In this paper, we report the crystal structure of the title compound, obtained by the reaction of *o*-phenylenediamine and 3-*tert*-butyl-2-hydroxybenzaldehyde.

The title compound (Fig. 1) has a slightly twisted geometry with the dihedral angles between the two benzene rings (C1–C6 and C15–C20) with the central benzene ring (C8–C13) being 29.12 (14) and 26.01 (14)°. The geometrical parameters are comparable to previously reported structures (Eltayeb *et al.*, 2007*a*,b). Two bifurcated intramolecular C—H···O hydrogen bonds and two intramolecular O—H···N hydrogen bonds stabilized the molecular structure (Fig. 1, Table 1). In the crystal packing (Fig. 2), the molecules are stacked along the *a* axis and stabilized by *Cg*1···*Cg*1 = 3.6724 (17) Å interactions [*Cg*1 is the centroid of C1–C6 phenyl ring; 1 - *x*, -*y*, 1 - *z*].

### Experimental

To a solution of *o*-phenylenediamine (0.216 g, 2 mmol) in ethanol (20 ml) was added 3-*tert*-butyl-2-hydroxybenzaldehyde (0.7 ml, 4 mmol). The mixture was refluxed with stirring for 30 min. The resultant orange solution was filtered. Yellow precipitate obtained was dissolved in acetone. Yellow crystals suitable for XRD formed after a few days of slow evaporation of the solvent at room temperature.

### Refinement

O-bound H atoms were located in a difference Fourier map and refined freely. The rest of the H atoms were positioned geometrically and refined using a riding model, with C–H = 0.93 or 0.96 Å and *U*_iso_(H) = 1.2–1.5(methyl)*U*_eq_(C). The rotating group model was applied for the methyl groups. The crystal studied was a non-merohedral twin with a refined BASF of 0.374 (5).

### Crystal data

**Table:**

C_28_H_32_N_2_O_2_	*Z* = 2
*M**_r_* = 428.56	*F*(000) = 460
Triclinic, *P*1	*D*_x_ = 1.238 Mg m^−^^3^
Hall symbol: -P 1	Mo *K*α radiation, λ = 0.71073 Å
*a* = 6.8312 (9) Å	Cell parameters from 4325 reflections
*b* = 13.9632 (16) Å	θ = 2.9–29.9°
*c* = 14.0689 (15) Å	µ = 0.08 mm^−^^1^
α = 116.615 (5)°	*T* = 100 K
β = 99.068 (4)°	Plate, yellow
γ = 98.209 (4)°	0.87 × 0.20 × 0.05 mm
*V* = 1149.6 (2) Å^3^	

### Data collection

**Table:**

Bruker SMART APEXII CCD area-detector diffractometer	4021 independent reflections
Radiation source: fine-focus sealed tube	3241 reflections with *I* > 2σ(*I*)
graphite	*R*_int_ = 0.0000
φ and ω scans	θ_max_ = 25.0°, θ_min_ = 1.7°
Absorption correction: multi-scan (*SADABS*; Bruker, 2005)	*h* = −8→7
*T*_min_ = 0.936, *T*_max_ = 0.996	*k* = −16→14
4021 measured reflections	*l* = −4→16

### Refinement

**Table:**

Refinement on *F*^2^	Primary atom site location: structure-invariant direct methods
Least-squares matrix: full	Secondary atom site location: difference Fourier map
*R*[*F*^2^ > 2σ(*F*^2^)] = 0.059	Hydrogen site location: inferred from neighbouring sites
*wR*(*F*^2^) = 0.206	H atoms treated by a mixture of independent and constrained refinement
*S* = 1.19	*w* = 1/[σ^2^(*F*_o_^2^) + (0.0995*P*)^2^ + 0.5885*P*] where *P* = (*F*_o_^2^ + 2*F*_c_^2^)/3
4021 reflections	(Δ/σ)_max_ < 0.001
304 parameters	Δρ_max_ = 0.32 e Å^−^^3^
0 restraints	Δρ_min_ = −0.39 e Å^−^^3^

### Special details

**Table:**

Experimental. The crystal was placed in the cold stream of an Oxford Cyrosystems Cobra open-flow nitrogen cryostat (Cosier & Glazer, 1986) operating at 100.0 (1) K.
Geometry. All e.s.d.'s (except the e.s.d. in the dihedral angle between two l.s. planes) are estimated using the full covariance matrix. The cell e.s.d.'s are taken into account individually in the estimation of e.s.d.'s in distances, angles and torsion angles; correlations between e.s.d.'s in cell parameters are only used when they are defined by crystal symmetry. An approximate (isotropic) treatment of cell e.s.d.'s is used for estimating e.s.d.'s involving l.s. planes.
Refinement. Refinement of *F*^2^ against ALL reflections. The weighted *R*-factor *wR* and goodness of fit *S* are based on *F*^2^, conventional *R*-factors *R* are based on *F*, with *F* set to zero for negative *F*^2^. The threshold expression of *F*^2^ > σ(*F*^2^) is used only for calculating *R*-factors(gt) *etc*. and is not relevant to the choice of reflections for refinement. *R*-factors based on *F*^2^ are statistically about twice as large as those based on *F*, and *R*- factors based on ALL data will be even larger.

### Fractional atomic coordinates and isotropic or equivalent isotropic displacement parameters (Å^2^)

**Table:**

	*x*	*y*	*z*	*U*_iso_*/*U*_eq_	
O1	0.2350 (3)	0.09518 (15)	0.37885 (15)	0.0209 (5)	
O2	−0.1083 (3)	0.11123 (16)	0.22061 (15)	0.0231 (5)	
N1	0.0669 (3)	−0.11626 (17)	0.27055 (17)	0.0176 (5)	
N2	−0.1856 (3)	−0.10123 (17)	0.10607 (18)	0.0177 (5)	
C1	0.2757 (4)	0.0859 (2)	0.4710 (2)	0.0173 (6)	
C2	0.3650 (4)	0.1817 (2)	0.5735 (2)	0.0180 (6)	
C3	0.4083 (4)	0.1661 (2)	0.6647 (2)	0.0205 (6)	
H3A	0.4711	0.2275	0.7327	0.025*	
C4	0.3624 (4)	0.0631 (2)	0.6593 (2)	0.0213 (6)	
H4A	0.3953	0.0565	0.7225	0.026*	
C5	0.2682 (4)	−0.0287 (2)	0.5601 (2)	0.0185 (6)	
H5A	0.2332	−0.0975	0.5562	0.022*	
C6	0.2250 (4)	−0.0188 (2)	0.4652 (2)	0.0166 (6)	
C7	0.1138 (4)	−0.1159 (2)	0.3629 (2)	0.0178 (6)	
H7A	0.0740	−0.1818	0.3641	0.021*	
C8	−0.0515 (4)	−0.2150 (2)	0.1771 (2)	0.0164 (6)	
C9	−0.0387 (4)	−0.3197 (2)	0.1618 (2)	0.0209 (6)	
H9A	0.0541	−0.3258	0.2136	0.025*	
C10	−0.1613 (5)	−0.4144 (2)	0.0713 (2)	0.0246 (7)	
H10A	−0.1506	−0.4834	0.0624	0.029*	
C11	−0.3008 (5)	−0.4060 (2)	−0.0064 (2)	0.0239 (7)	
H11A	−0.3866	−0.4694	−0.0663	0.029*	
C12	−0.3115 (4)	−0.3039 (2)	0.0056 (2)	0.0211 (6)	
H12A	−0.4059	−0.2991	−0.0465	0.025*	
C13	−0.1838 (4)	−0.2070 (2)	0.0942 (2)	0.0174 (6)	
C14	−0.2331 (4)	−0.0902 (2)	0.0202 (2)	0.0191 (6)	
H14A	−0.2692	−0.1530	−0.0484	0.023*	
C15	−0.2329 (4)	0.0154 (2)	0.0251 (2)	0.0181 (6)	
C16	−0.2928 (4)	0.0202 (2)	−0.0721 (2)	0.0218 (6)	
H16A	−0.3379	−0.0447	−0.1389	0.026*	
C17	−0.2855 (4)	0.1196 (2)	−0.0698 (2)	0.0232 (6)	
H17A	−0.3291	0.1223	−0.1344	0.028*	
C18	−0.2127 (4)	0.2166 (2)	0.0297 (2)	0.0222 (6)	
H18A	−0.2052	0.2835	0.0296	0.027*	
C19	−0.1506 (4)	0.2182 (2)	0.1293 (2)	0.0202 (6)	
C20	−0.1651 (4)	0.1147 (2)	0.1261 (2)	0.0187 (6)	
C21	0.4042 (4)	0.2968 (2)	0.5821 (2)	0.0218 (6)	
C22	0.2009 (4)	0.3178 (2)	0.5415 (2)	0.0241 (6)	
H22A	0.1358	0.2601	0.4681	0.036*	
H22B	0.2262	0.3876	0.5421	0.036*	
H22C	0.1134	0.3191	0.5890	0.036*	
C23	0.5547 (5)	0.3065 (2)	0.5150 (2)	0.0265 (7)	
H23A	0.5013	0.2504	0.4397	0.040*	
H23B	0.6834	0.2972	0.5439	0.040*	
H23C	0.5739	0.3779	0.5194	0.040*	
C24	0.4951 (5)	0.3884 (2)	0.7013 (2)	0.0271 (7)	
H24A	0.6252	0.3795	0.7290	0.041*	
H24B	0.4044	0.3838	0.7456	0.041*	
H24C	0.5125	0.4591	0.7040	0.041*	
C25	−0.0608 (5)	0.3263 (2)	0.2379 (2)	0.0237 (7)	
C26	−0.1878 (5)	0.3344 (2)	0.3208 (3)	0.0306 (7)	
H26A	−0.1278	0.4014	0.3887	0.046*	
H26B	−0.1901	0.2723	0.3336	0.046*	
H26C	−0.3248	0.3346	0.2921	0.046*	
C27	0.1622 (5)	0.3313 (2)	0.2836 (2)	0.0263 (7)	
H27A	0.2176	0.3976	0.3526	0.039*	
H27B	0.2406	0.3311	0.2326	0.039*	
H27C	0.1676	0.2683	0.2941	0.039*	
C28	−0.0596 (5)	0.4277 (2)	0.2213 (3)	0.0299 (7)	
H28A	−0.0024	0.4936	0.2905	0.045*	
H28B	−0.1970	0.4272	0.1922	0.045*	
H28C	0.0213	0.4258	0.1708	0.045*	
H1O2	−0.127 (6)	0.038 (3)	0.199 (3)	0.049 (11)*	
H1O1	0.174 (7)	0.026 (4)	0.322 (4)	0.064 (13)*	

### Atomic displacement parameters (Å^2^)

**Table:**

	*U*^11^	*U*^22^	*U*^33^	*U*^12^	*U*^13^	*U*^23^
O1	0.0261 (11)	0.0191 (10)	0.0153 (9)	0.0031 (8)	0.0029 (8)	0.0079 (8)
O2	0.0313 (12)	0.0210 (10)	0.0194 (10)	0.0070 (9)	0.0042 (9)	0.0120 (8)
N1	0.0133 (12)	0.0176 (11)	0.0192 (12)	0.0036 (9)	0.0042 (9)	0.0066 (9)
N2	0.0142 (12)	0.0192 (11)	0.0206 (12)	0.0052 (9)	0.0042 (9)	0.0100 (9)
C1	0.0108 (13)	0.0255 (14)	0.0159 (13)	0.0061 (11)	0.0040 (10)	0.0095 (11)
C2	0.0105 (13)	0.0226 (14)	0.0186 (13)	0.0040 (11)	0.0057 (11)	0.0073 (11)
C3	0.0140 (14)	0.0250 (14)	0.0155 (13)	0.0033 (11)	0.0043 (11)	0.0040 (11)
C4	0.0170 (14)	0.0312 (15)	0.0175 (13)	0.0082 (12)	0.0051 (11)	0.0124 (12)
C5	0.0129 (14)	0.0247 (14)	0.0210 (14)	0.0081 (11)	0.0061 (11)	0.0120 (12)
C6	0.0088 (13)	0.0213 (13)	0.0201 (13)	0.0059 (10)	0.0053 (11)	0.0091 (11)
C7	0.0149 (14)	0.0201 (13)	0.0200 (14)	0.0065 (11)	0.0060 (11)	0.0098 (11)
C8	0.0130 (13)	0.0161 (13)	0.0179 (13)	0.0022 (10)	0.0063 (11)	0.0059 (11)
C9	0.0230 (15)	0.0227 (14)	0.0197 (14)	0.0091 (12)	0.0070 (12)	0.0110 (12)
C10	0.0336 (17)	0.0151 (13)	0.0249 (15)	0.0064 (12)	0.0109 (13)	0.0083 (12)
C11	0.0262 (16)	0.0192 (14)	0.0178 (14)	−0.0017 (12)	0.0055 (12)	0.0038 (11)
C12	0.0182 (14)	0.0250 (14)	0.0179 (13)	0.0028 (11)	0.0039 (11)	0.0094 (11)
C13	0.0136 (13)	0.0196 (13)	0.0185 (13)	0.0037 (11)	0.0073 (11)	0.0078 (11)
C14	0.0119 (13)	0.0232 (14)	0.0187 (13)	0.0024 (11)	0.0033 (11)	0.0079 (11)
C15	0.0096 (13)	0.0277 (14)	0.0202 (13)	0.0060 (11)	0.0044 (11)	0.0135 (12)
C16	0.0131 (14)	0.0320 (15)	0.0196 (14)	0.0044 (12)	0.0051 (11)	0.0117 (12)
C17	0.0135 (14)	0.0402 (16)	0.0265 (15)	0.0080 (12)	0.0063 (12)	0.0239 (13)
C18	0.0181 (14)	0.0298 (15)	0.0296 (15)	0.0107 (12)	0.0102 (12)	0.0206 (13)
C19	0.0142 (14)	0.0263 (14)	0.0264 (15)	0.0085 (11)	0.0091 (11)	0.0155 (12)
C20	0.0142 (14)	0.0270 (14)	0.0204 (14)	0.0077 (11)	0.0066 (11)	0.0146 (12)
C21	0.0194 (15)	0.0212 (14)	0.0217 (14)	0.0041 (11)	0.0068 (12)	0.0075 (12)
C22	0.0253 (16)	0.0233 (14)	0.0261 (15)	0.0075 (12)	0.0067 (13)	0.0133 (12)
C23	0.0259 (16)	0.0224 (14)	0.0274 (15)	0.0021 (12)	0.0087 (13)	0.0092 (12)
C24	0.0244 (16)	0.0227 (15)	0.0251 (15)	0.0022 (12)	0.0055 (13)	0.0052 (12)
C25	0.0277 (17)	0.0226 (14)	0.0257 (15)	0.0091 (12)	0.0104 (13)	0.0137 (12)
C26	0.0370 (19)	0.0291 (16)	0.0318 (16)	0.0139 (14)	0.0166 (15)	0.0156 (14)
C27	0.0267 (16)	0.0241 (14)	0.0245 (15)	0.0036 (12)	0.0049 (13)	0.0100 (12)
C28	0.0356 (18)	0.0275 (15)	0.0342 (17)	0.0111 (14)	0.0140 (14)	0.0185 (14)

### Geometric parameters (Å, °)

**Table:**

O1—C1	1.349 (3)	C15—C20	1.413 (4)
O1—H1O1	0.91 (4)	C16—C17	1.368 (4)
O2—C20	1.350 (3)	C16—H16A	0.9300
O2—H1O2	0.91 (4)	C17—C18	1.389 (4)
N1—C7	1.286 (3)	C17—H17A	0.9300
N1—C8	1.416 (3)	C18—C19	1.387 (4)
N2—C14	1.284 (3)	C18—H18A	0.9300
N2—C13	1.412 (3)	C19—C20	1.414 (4)
C1—C2	1.415 (4)	C19—C25	1.538 (4)
C1—C6	1.416 (4)	C21—C23	1.531 (4)
C2—C3	1.389 (4)	C21—C24	1.536 (4)
C2—C21	1.537 (4)	C21—C22	1.536 (4)
C3—C4	1.393 (4)	C22—H22A	0.9600
C3—H3A	0.9300	C22—H22B	0.9600
C4—C5	1.372 (4)	C22—H22C	0.9600
C4—H4A	0.9300	C23—H23A	0.9600
C5—C6	1.395 (4)	C23—H23B	0.9600
C5—H5A	0.9300	C23—H23C	0.9600
C6—C7	1.449 (4)	C24—H24A	0.9600
C7—H7A	0.9300	C24—H24B	0.9600
C8—C9	1.397 (4)	C24—H24C	0.9600
C8—C13	1.416 (4)	C25—C28	1.534 (4)
C9—C10	1.383 (4)	C25—C26	1.535 (4)
C9—H9A	0.9300	C25—C27	1.536 (4)
C10—C11	1.391 (4)	C26—H26A	0.9600
C10—H10A	0.9300	C26—H26B	0.9600
C11—C12	1.373 (4)	C26—H26C	0.9600
C11—H11A	0.9300	C27—H27A	0.9600
C12—C13	1.398 (4)	C27—H27B	0.9600
C12—H12A	0.9300	C27—H27C	0.9600
C14—C15	1.445 (4)	C28—H28A	0.9600
C14—H14A	0.9300	C28—H28B	0.9600
C15—C16	1.399 (4)	C28—H28C	0.9600
			
C1—O1—H1O1	107 (3)	C19—C18—H18A	118.5
C20—O2—H1O2	104 (2)	C17—C18—H18A	118.5
C7—N1—C8	118.6 (2)	C18—C19—C20	116.7 (2)
C14—N2—C13	119.5 (2)	C18—C19—C25	122.3 (2)
O1—C1—C2	119.7 (2)	C20—C19—C25	120.9 (2)
O1—C1—C6	120.1 (2)	O2—C20—C15	119.8 (2)
C2—C1—C6	120.3 (2)	O2—C20—C19	119.3 (2)
C3—C2—C1	116.8 (2)	C15—C20—C19	120.9 (2)
C3—C2—C21	122.5 (2)	C23—C21—C24	107.6 (2)
C1—C2—C21	120.7 (2)	C23—C21—C22	110.5 (2)
C2—C3—C4	123.2 (2)	C24—C21—C22	106.9 (2)
C2—C3—H3A	118.4	C23—C21—C2	110.6 (2)
C4—C3—H3A	118.4	C24—C21—C2	111.7 (2)
C5—C4—C3	119.5 (3)	C22—C21—C2	109.4 (2)
C5—C4—H4A	120.2	C21—C22—H22A	109.5
C3—C4—H4A	120.2	C21—C22—H22B	109.5
C4—C5—C6	119.9 (2)	H22A—C22—H22B	109.5
C4—C5—H5A	120.1	C21—C22—H22C	109.5
C6—C5—H5A	120.0	H22A—C22—H22C	109.5
C5—C6—C1	120.2 (2)	H22B—C22—H22C	109.5
C5—C6—C7	118.6 (2)	C21—C23—H23A	109.5
C1—C6—C7	121.0 (2)	C21—C23—H23B	109.5
N1—C7—C6	123.8 (2)	H23A—C23—H23B	109.5
N1—C7—H7A	118.1	C21—C23—H23C	109.5
C6—C7—H7A	118.1	H23A—C23—H23C	109.5
C9—C8—C13	118.7 (2)	H23B—C23—H23C	109.5
C9—C8—N1	122.9 (2)	C21—C24—H24A	109.5
C13—C8—N1	118.4 (2)	C21—C24—H24B	109.5
C10—C9—C8	121.3 (3)	H24A—C24—H24B	109.5
C10—C9—H9A	119.4	C21—C24—H24C	109.5
C8—C9—H9A	119.4	H24A—C24—H24C	109.5
C9—C10—C11	119.7 (2)	H24B—C24—H24C	109.5
C9—C10—H10A	120.1	C28—C25—C26	107.5 (2)
C11—C10—H10A	120.1	C28—C25—C27	107.1 (2)
C12—C11—C10	119.8 (3)	C26—C25—C27	110.9 (2)
C12—C11—H11A	120.1	C28—C25—C19	111.6 (2)
C10—C11—H11A	120.1	C26—C25—C19	110.5 (2)
C11—C12—C13	121.6 (3)	C27—C25—C19	109.2 (2)
C11—C12—H12A	119.2	C25—C26—H26A	109.5
C13—C12—H12A	119.2	C25—C26—H26B	109.5
C12—C13—N2	122.8 (2)	H26A—C26—H26B	109.5
C12—C13—C8	118.6 (2)	C25—C26—H26C	109.5
N2—C13—C8	118.6 (2)	H26A—C26—H26C	109.5
N2—C14—C15	122.9 (2)	H26B—C26—H26C	109.5
N2—C14—H14A	118.5	C25—C27—H27A	109.5
C15—C14—H14A	118.5	C25—C27—H27B	109.5
C16—C15—C20	119.2 (2)	H27A—C27—H27B	109.5
C16—C15—C14	119.5 (2)	C25—C27—H27C	109.5
C20—C15—C14	121.3 (2)	H27A—C27—H27C	109.5
C17—C16—C15	120.5 (3)	H27B—C27—H27C	109.5
C17—C16—H16A	119.8	C25—C28—H28A	109.5
C15—C16—H16A	119.8	C25—C28—H28B	109.5
C16—C17—C18	119.6 (3)	H28A—C28—H28B	109.5
C16—C17—H17A	120.2	C25—C28—H28C	109.5
C18—C17—H17A	120.2	H28A—C28—H28C	109.5
C19—C18—C17	123.1 (3)	H28B—C28—H28C	109.5
			
O1—C1—C2—C3	−178.2 (2)	N1—C8—C13—N2	2.6 (4)
C6—C1—C2—C3	3.2 (4)	C13—N2—C14—C15	−178.2 (2)
O1—C1—C2—C21	3.7 (4)	N2—C14—C15—C16	−178.0 (3)
C6—C1—C2—C21	−174.9 (2)	N2—C14—C15—C20	4.8 (4)
C1—C2—C3—C4	−2.1 (4)	C20—C15—C16—C17	0.1 (4)
C21—C2—C3—C4	176.0 (2)	C14—C15—C16—C17	−177.2 (2)
C2—C3—C4—C5	−0.5 (4)	C15—C16—C17—C18	1.8 (4)
C3—C4—C5—C6	2.0 (4)	C16—C17—C18—C19	−1.8 (4)
C4—C5—C6—C1	−0.8 (4)	C17—C18—C19—C20	−0.2 (4)
C4—C5—C6—C7	−176.1 (2)	C17—C18—C19—C25	177.0 (3)
O1—C1—C6—C5	179.5 (2)	C16—C15—C20—O2	179.5 (2)
C2—C1—C6—C5	−1.9 (4)	C14—C15—C20—O2	−3.2 (4)
O1—C1—C6—C7	−5.3 (4)	C16—C15—C20—C19	−2.2 (4)
C2—C1—C6—C7	173.3 (2)	C14—C15—C20—C19	175.1 (2)
C8—N1—C7—C6	−176.7 (2)	C18—C19—C20—O2	−179.5 (2)
C5—C6—C7—N1	−179.6 (3)	C25—C19—C20—O2	3.2 (4)
C1—C6—C7—N1	5.2 (4)	C18—C19—C20—C15	2.2 (4)
C7—N1—C8—C9	−32.3 (4)	C25—C19—C20—C15	−175.1 (2)
C7—N1—C8—C13	149.7 (2)	C3—C2—C21—C23	120.0 (3)
C13—C8—C9—C10	−4.1 (4)	C1—C2—C21—C23	−62.0 (3)
N1—C8—C9—C10	177.9 (2)	C3—C2—C21—C24	0.1 (4)
C8—C9—C10—C11	−0.1 (4)	C1—C2—C21—C24	178.1 (2)
C9—C10—C11—C12	2.0 (4)	C3—C2—C21—C22	−118.1 (3)
C10—C11—C12—C13	0.5 (4)	C1—C2—C21—C22	59.9 (3)
C11—C12—C13—N2	177.3 (2)	C18—C19—C25—C28	2.6 (4)
C11—C12—C13—C8	−4.7 (4)	C20—C19—C25—C28	179.7 (2)
C14—N2—C13—C12	−29.9 (4)	C18—C19—C25—C26	122.2 (3)
C14—N2—C13—C8	152.0 (3)	C20—C19—C25—C26	−60.7 (3)
C9—C8—C13—C12	6.4 (4)	C18—C19—C25—C27	−115.6 (3)
N1—C8—C13—C12	−175.6 (2)	C20—C19—C25—C27	61.5 (3)
C9—C8—C13—N2	−175.5 (2)		

### Hydrogen-bond geometry (Å, °)

**Table:**

*D*—H···*A*	*D*—H	H···*A*	*D*···*A*	*D*—H···*A*
O2—H1O2···N2	0.91 (5)	1.73 (4)	2.584 (3)	156 (4)
O1—H1O1···N1	0.91 (5)	1.77 (6)	2.609 (3)	151 (5)
C22—H22A···O1	0.96	2.34	2.993 (3)	125
C23—H23A···O1	0.96	2.34	2.987 (4)	124
C26—H26B···O2	0.96	2.31	2.963 (4)	125
C27—H27C···O2	0.96	2.37	3.011 (4)	124
